# COVID-19 therapeutics: how to sow confusion and break public trust during international public health emergencies

**DOI:** 10.1186/s40545-020-00244-0

**Published:** 2020-07-24

**Authors:** Jerome Amir Singh, Rafaella Ravinetto

**Affiliations:** 1 Centre for the AIDS Programme of Research in South Africa (CAPRISA), University of KwaZulu-Natal, Durban, South Africa; 2 Institute for Tropical Medicine, Antwerp, Belgium

## Abstract

Since SARS-CoV2 was declared a *Public Health Emergency of International Concern*, those tasked with the stewardship of public health at a global, regional, and local level—policymakers, politicians, scientists, drug regulators, health officials, professional associations, journal editors, publishers, and clinicians—have displayed rushed decisions and lapses in judgment in their handling of chloroquine and hydroxychloroquine as potential COVID-19 therapeutics and prophylactics. These lapses merit noting as they hold lessons for how the guardians of medicines regulation and public health can inadvertently sow confusion and damage public trust.

## Background

The novel coronavirus SARS-CoV-2 represents a significant and urgent threat to global health. With the global tally of infected people now numbering in the millions, and the number of people who have succumbed to the disease now quantified in the hundreds of thousands, the identification of efficacious SARS CoV-2 therapeutic candidates has quickly become one of the world’s most pressing health needs. Since SARS-CoV2 was declared a *Public Health Emergency of International Concern*, those tasked with the stewardship of public health at a global, regional, and local level—drug regulators, policymakers, politicians, scientists, health officials, professional associations, journal editors, publishers, and clinicians—have displayed rushed decisions and lapses in judgment in their handling of chloroquine (CQ) and hydroxychloroquine (HCQ) as potential COVID-19 therapeutics and prophylactics. These lapses merit noting as they hold lessons for how the guardians of medicines regulation and public health can inadvertently sow confusion and damage public trust. Such outcomes are antithetical to what the world needs when it is confronting a serious public health emergency of international concern.

## A review of key global developments on CQ and HCQ in the context of COVID-19

In February 2020, Chinese scientists reported that CQ had demonstrated apparent efficacy and acceptable safety against COVID-19 associated pneumonia in multi-centre clinical trials conducted in China [1]. On 13 March 2020, the WHO published interim guidance for the management of COVID-19 [2]. The guidance contained no recommendation in regard to CQ or HCQ. On 16 March 2020, a French researcher announced early results of an open-label, single-group study that involved contemporaneous, but nonrandomized controls, which indicated that HCQ treatment was significantly associated with viral load reduction in COVID-19 patients, and the results were published days later in the *International Journal of Antimicrobial Agents* (*IJAA*) [3, 4]. On 18 March 2020, to expedite the identification of efficacious candidate agents, the WHO and partners launched the ‘Solidarity Trial’, an international randomised clinical trial designed to compare four treatment options for hospitalised patients—remdesivir; lopinavir/ritonavir; lopinavir/ritonavir with interferon beta-1a; and CQ or HCQ—against standard of care, to assess their relative effectiveness against COVID-19 [5]. On 19 March 2020, researchers in the UK launched the Randomised Evaluation of COVid-19 thERapY (RECOVERY) Trial, billed as the world’s largest randomised clinical trial of potential coronavirus treatments (including HCQ) in a hospital setting [6], following the trial’s approval on 17 March 2020 [7]. Following news coverage in the USA of the earlier announced French study [8], by 19 March 2020, the US President, Donald Trump, began hailing the drug as a ‘game changer’ against COVID-19 [9]. Trump’s touting triggered a surge in demand, hoarding, and associated shortages for those who needed the drug for lupus or rheumatoid arthritis [10], and even deaths [11]. By 21 March 2020, the US Centres for Disease Control (US CDC), under pressure from the US administration, published guidance on dosing information on HCQ and CQ based on unattributed anecdotes rather than peer-reviewed science [12]. On 21 March 2020, Brazil’s president, Jair Bolsanaro, echoed Trump’s support for the drug as a cure for COVID-19 [13]. Such high-profile political support precipitated unprecedented demand and shortages in countries such as the UK, France, Thailand, India, and Australia, prompting cautionary advisories by some professional associations [14] and regulatory authorities [15] against the off-label use of these drugs. Under pressure from the French researchers who ran the small trial published in the *IJAA* that suggested that HCQ was associated with COVID-19 viral load reduction [16], on 23 March 2020, the French Health Minister announced that in the absence of any conclusive data, he would be issuing an order to ‘regulate the use of hydroxychloroquine outside the traditional marketing authorisations, which will therefore be accessible to hospital medical teams who wish to use it’ [17]. Further, that while the country’s High Council of Public Health had not recommended using HCQ ‘in the absence of a recommendation’, the Council made an exception for ‘serious forms of hospitalisation and on the collegial decision of doctors and under strict medical supervision’ [18]. On 25 March 2020, the UK’s drug regulator, the Medicines and Healthcare products Regulatory Agency (MHRA), took the opposite stance, cautioning that CQ and HCQ were not licensed to treat COVID-19 related symptoms or prevent infection and that until there was clear, definitive evidence from ongoing clinical trials that both drugs were safe and effective for the treatment of COVID-19, they should only be used for this purpose within a clinical trial [19]. On 27 March 2020, Italy announcing that CQ and HCQ could be used to treat all COVID-19 patients and would be fully sponsored by the Italian national healthcare system [16]. Despite the use of CQ and HCQ for COVID-19 being described as ‘egregious’ and ‘misguided at best and dangerous at worst’ by members of the US scientific community [20], on 28 March 2020, the US Food and Drug Administration (FDA) issued an ‘Emergency Use Authorization’ to allow HCQ and CQ to be distributed and used for certain hospitalized patients with COVID-19 [21]. Since then, dozens more studies were initiated globally to determine the treatment, post-exposure prophylactic, and prophylactic, efficacy of CQ and HCQ against COVID-19.

On 1 April 2020, while acknowledging that urgency and pressures on their health systems had prompted countries such as France and the USA to put strict protocols in place to allow for the use of CQ and HCQ against COVID-19, the European Medicine Agency (EMA) cautioned that the efficacy of CQ and HCQ against COVID-19 had yet to be established in studies, and that the drugs should only be utilised for their authorised uses or as part of clinical trials or national emergency use programmes for the treatment of COVID-19 [22]. On 1 April 2020, South Africa’s drug regulatory authority, the South African Health Products Regulatory Authority (SAPHRA), which had not too long before then approved the Solidarity Trial in South Africa, authorised the import of half a million chloroquine phosphate tablets for use in severely ill COVID-19 patients, at the behest of the country’s national Department of Health and a pharmaceutical company [23]. With these divergent approaches breeding confusion, on 2 April 2020, the International Coalition of Medicines Regulatory Authorities (ICMRA) convened a virtual meeting of experts from leading national medicines regulatory authorities, the World Health Organization (WHO), and the European Commission [24]. Participants of the meeting acknowledged that small studies or compassionate use programmes are unlikely to be able to generate the required level of evidence to allow clear-cut recommendations. Nevertheless, global regulators concurred that ‘compassionate use programmes, which allow access to potential therapies for patients in need, have a beneficial public health impact on the pandemic and should be allowed, as long as they do not pose a threat to clinical trials recruitment’. On 3 April 2020, the International Society of Antimicrobial Chemotherapy (ISAC), the society that publishes the *IJAA*, the journal in which the small French study that initially reported HCQ efficacy against COVID-19, issued a statement noting that it ‘shared the concerns’ regarding the article [25]. Further, that the ISAC Board believed the article ‘[did] not meet the Society’s expected standard, especially relating to the lack of better explanations of the inclusion criteria and the triage of patients to ensure patient safety’. The ISAC noted that although it ‘recognises it is important to help the scientific community by publishing new data fast, this cannot be at the cost of reducing scientific scrutiny and best practices’. On the hand, the *IJAA* itself failed to do the same. On 8 April 2020, some US professional associations cautioned against the use of CQ and HCQ for the treatment of COVID-19 [26]. On 8 April 2020, the Pharmaceutical Society of Kenya issued a statement recommending that health care providers use the WHO recommendations for management of COVID-19 patients and discouraging over the counter dispensing of HCQ by community pharmacists [27]. On 21 April 2020, the Ugandan government announced that it was deploying HCQ as a treatment regimen [28]. On 24 April 2020, the US FDA issued a drug safety communication regarding known side effects of HCQ and CQ, including serious and potentially life-threatening heart rhythm problems, that had been reported with their off-label use for the treatment or prevention of COVID-19 [29, 30]. On 24 April 2020, divergent approaches on the part of African governments and drug regulators prompted the African Centres for Disease Control (CDC) to advise ‘Physicians should not prescribe, and individuals should not take, chloroquine or hydroxychloroquine to prevent or treat COVID-19 except under clinical trial or monitored emergency use of unregistered and investigational interventions (MEURI). These drugs can cause neurologic, ophthalmic, cardiac, and other forms of toxicity. Physicians should not prescribe, and individuals should not take, Lopinavir/Ritonavir, Remdesivir or other medications to prevent or treat COVID-19 except under clinical trial or MEURI’ [31].

Despite the African CDC’s advisory caution, by 1 May 2020, Kenya ordered a one-off consignment of 379,000 HCQ tablets from India, 3 weeks after India partially lifted its ban on the export of the drug [32]. In so doing, Kenya joined the growing list of countries that had repurposed HCQ for emergency use against COVID-19. On 15 May 2020, the UK government put a tender for pharmaceutical suppliers to supply more than 33 m tablets of various drugs between June 2020 and January 2021, including 16 million tablets of HCQ, for use as COVID-19 therapeutics [33]. This, despite the country’s drug regulatory authority, the MHRA, cautioning against the use of both drugs outside of clinical trials [19]. On 21 May 2020, reports emerged that 10 million doses (> 5 t) of CQ had been illegally imported into South Africa, seemingly for use against COVID-19 [34]. On 22 May 2020, *The Lancet* published the results of an observational study based on approximately 96,000 patients hospitalized between December 2019 and April 2020 in 671 hospitals, which suggested that HCQ or CQ, when used alone or with a macrolide, was associated with decreased in-hospital survival and an increased frequency of ventricular arrhythmias when used for treatment of COVID-19 [35]. Following this publication, on 23 May 2020, the Executive Group of the International Steering Committee of WHO’s Solidarity Trial implemented a temporary pause of the trial’s HQ arm while the safety data was reviewed by the study’s Data Safety Monitoring Board (DSMB). The study’s Executive Group also decided to conduct a comprehensive analysis and critical appraisal of all evidence available globally on HCQ [36]. By then, over 400 hospitals in 35 countries were actively recruiting patients, and nearly 3500 patients had been enrolled from 17 countries. Based on *The Lancet* trial’s findings, on 22 May 2020, the UK’s drug regulatory body, the MHRA, notified the chief investigators of the RECOVERY Trial of their concerns relating to the use of HCQ as a treatment for patients with COVID-19 [37]. On 24 May 2020, the RECOVERY trial’s chief investigators announced that the trial’s independent Data Monitoring Committee (DMC) had conducted an urgent review of unblinded safety data for the HCQ arms of the trial and saw no reason to suspend recruitment. As a result, the MHRA concluded that it was acceptable to continue the HCQ arm of the trial. Meanwhile, countries such as France, Italy, and Australia suspended the inclusion of new participants in clinical trials testing the efficacy of HCQ and/or revoked the authorization allowing HCQ to be used as off-label treatment for COVID-19 patients [38–40]. On 26 May 2020, the French High Council of Public Health (HCSP) recommended against the use of HCQ (alone or in combination with a macrolide) for the treatment of COVID-19 [38]. Despite these developments, the presidents of the USA, Brazil, and El Salvadore continued to punt CQ/HCQ for the use as treatment (and prevention) against COVID-19 [41, 42]. On 26 May 2020, South African investigators of the COVID-19 Research Outcomes Worldwide Network (CROWN) study, a large US-led international study aimed at assessing the efficacy of CQ for preventing COVID-19 or decreasing the severity of infection in front-line healthcare workers [43], announced that they had obtained local ethics approval to commence the trial in South Africa, and that as *The Lancet* study’s data did not apply to the use of any drug regimen used in the ambulatory, out-of-hospital setting, the trial would go ahead in South Africa [44]. On 28 May 2020, 120 researchers signed a letter to *The Lancet,* highlighting concerns about the quality of the data and its analysis of the HCQ study published in the journal on 22 May 2020 [45]. On 31 May 2020, the US government announced that it had shipped 2 million doses of HCQ to Brazil as a humanitarian gesture for therapeutic and prophylactic use against COVID-19 [46].

On 2 June 2020, *The Lancet* issued an ‘Expression of Concern’ to alert readers that ‘serious scientific questions’ had been brought to its attention regarding the HCQ study published in the journal on 22 May 2020 [47]. France’s drug regulatory agency, L’Agence nationale de sécurité du médicament et des produits de santé/Agency for the Safety of Medicines and Health Products (ANSM), announced that it had suspended the inclusion of new participants in clinical trials testing the efficacy of HCQ in France, although patients already being treated with HCQ could continue their treatment regimen until the end of their treatment protocol. On 4 June 2020, the WHO announced that the Solidarity Trial’s DSMB reviewed available mortality data and recommended that there were no reasons to modify the trial protocol [48]. Accordingly, the study’s Executive Group endorsed the continuation of the HCQ arm of the trial. On 5 June 2020, some of the authors of *The Lancet* paper retracted the paper because they were unable to complete an independent audit of the data underpinning their analysis [49]. On 5 June 2020, the chief investigators of the RECOVERY Trial announced that the study’s DMC conducted a further review of study data. Based thereon, the DMC found that HCQ demonstrated no mortality benefit for patients hospitalised with COVID-19 [50]. Accordingly, the investigators ceased the enrolment of participants to the trial’s HCQ arm with immediate effect. On 15 June 2020, the US FDA revoked its ‘Emergency Use Authorization’ for chloroquine phosphate and hydroxychloroquine sulfate for the treatment of COVID-19 [51], holding that it is unlikely that CQ and HCQ may be effective in treating COVID-19, and that the known and potential benefits of CQ and HCQ do not outweigh the known and potential risks for the authorized uses. By then, the US federal government had distributed 31 million HCQ tablets to state and local health departments and had another 63 million HCQ tablets stockpiled, the future fate of which, is now uncertain [52]. On 17 June 2020, WHO announced that the HCQ arm of the Solidarity Trial to find an effective COVID-19 treatment for hospitalised patients was being stopped [52, 53]. It is unclear whether ongoing trials elsewhere that are evaluating the efficacy of varying doses of HCQ as treatment (with patients of varying morbidity) and prevention against COVID-19, in hospital and community settings, will follow suit [54]. Following *The Lancet* questioning whether the lack of transparency around the data of the retracted HCQ study it had published were the results of “fabrications” and “research misconduct”, and denying that there were lapses in its peer review process, the journal announced that it was changing its peer review process, by requiring both peer reviewers and authors to provide statements giving assurances on the integrity of data and methods in the paper being reviewed [55].

## Lessons learned

These dizzying arrays of global developments hold important lessons for the stewardship of public health emergencies of international concern. Because of the WHO’s perceived role as the steward of global health and its influence on decision-making on global health issues, its oversight committees need to be more circumspect in evaluating evidence of relevance to their ongoing trials as decision-making on major WHO trials will likely impact on policymaking and regulatory decision-making elsewhere. The results of the RECOVERY trial regarding HCQ highlight the importance of large, randomised trials to inform decisions about both the efficacy and the safety of treatments. While a cautionary approach is prudent, if observational study data is being relied upon, the data sources should be verified/audited before a decision is made about trial enrolment suspensions, regardless of the prominence of the journal the findings were published in.

Drug regulators should aim at coordinated decision-making, particularly when they belong to a same harmonization framework. Despite the European Medicine Agency cautioning that the efficacy of CQ and HCQ against COVID-19 had yet to be established in studies, and that the drugs should only be utilised for their authorised uses or as part of clinical trials or national emergency use programmes for the treatment of COVID-19 [22], member countries, such as France and Italy, authorised off-label use of HCQ for the treatment of COVID-19. The rapid and somewhat premature authorization of use of CQ and HCQ through concurrent but disparate regulatory and clinical pathways such as ‘off-label’ use, ‘compassionate use’, Monitored Emergency Use of Unregistered Interventions (MEURI), and ‘Emergency Use Authorisation’ undermines critical clinical trials being conducted to determine the efficacy of repurposed or investigational drugs. Such disparate actions also sow confusion amongst clinicians and the general public too. Notwithstanding urgency, risk assessment, pharmacovigilance and responsible governance still apply during public health emergencies.

Politicians should never ‘invade’ the field of competency of national regulators and peddle interventions of unproven efficacy. Doing so is irresponsible as it could trigger unnecessary stockpiling, shortages, illegal imports, and hoarding, thereby impacting on the treatment of patients who need the drug for other conditions. The promotion of potential therapeutics of unknown efficacy is also dangerous if it is later conclusively determined that the touted drugs are ineffective, or even linked to increased morbidity and/or mortality. At a bedside level, clinicians should not heed the unscientific touting of politicians. The rushed hoarding, prescription, and administration of candidate off-label therapeutics on the part of clinicians in the absence of evidence-based guidance—especially in a climate of fear—–carry the potential for abuse and misuse with consequences that might be worse than a user’s lack of access to that product. The off-label use of drugs such as CQ and HCQ for COVID-19 does not help answer questions about its safety and can also present a risk to the patient. Such outcomes could also erode the patient and public trust in clinicians and health systems.

Lastly, the scientific community needs to act responsibly in conducting and reviewing science. Public health emergencies do not justify diminished scientific rigor, and rapid review should not put at stake the quality of the review. While the investigators of *The Lancet* study claimed that they could not access databases to verify patient data, this reason is not defensible. While concealing identifiable individual patient data is important to protect patient privacy, the integrity of the data could have been verified in other ways. In any further investigation or audit of the study, the holders of the data sets should, at minimum, confirm that they have provided data [56]. Publishers and journals need to act fast and decisively when integrity issues arise. While rapid-pace research is crucial in a public health emergency, poor quality research and rash decision-making can impact on trust and ongoing research efforts. Knee-jerk reactions of health authorities send mixed signals to the general public. Some countries permitted, then suspended, the off-label use of HCQ by community-based clinicians. Rushed or uncoordinated decision-making risks undermining trust and confidence in scientists, health officials, and regulators.

## Conclusion

Times of crisis do not warrant premature, panic-driven decision-making, not supported by a solid risk assessment. Political leaders need to offer informed ethical advice, not peddle false hope and propaganda. Regulators need to guard their independence. The decisions of regulators and policymakers should be predicated on sound science, and they should not be a subject to political pressure or give in to populism. The duty of research sponsors and scientists is to adduce robust evidence, not hype. Scientific integrity must always supersede the pursuit of plaudits, publicity, prestige, and glory. Patient care and public health decision-making must be evidence-based, not hype-based. The world needs informed, responsible leadership if we are to manage the COVID-19 pandemic. If the stewards of global health lose the faith, confidence, and trust of the public, efforts to eradicate the COVID-19 pandemic will falter. The world cannot afford this outcome.

## Data Availability

Not applicable. All citations are publicly accessible.
